# 血清/血浆microRNAs作为肺癌诊断标记物的研究进展

**DOI:** 10.3779/j.issn.1009-3419.2012.01.11

**Published:** 2012-01-20

**Affiliations:** 200433 上海，同济大学附属上海市肺科医院胸外科 Department of Thoracic Surgery, Shanghai Pulmonary Hospital Affiliated to Tongji University, Shanghai 200433, China

近年来随着肺癌发病率与死亡率的逐年上升，肺癌已成为最具有致命性的恶性肿瘤之一。据美国癌症学会发布的一项数据显示，2010年美国确诊肺癌患者约22.2万人，约15.7万人死于肺癌，位居因恶性肿瘤死亡人数的首位^[1]^。在我国肺癌也已成为位居首位的癌症死因^[2]^。在所有肺癌中非小细胞肺癌（non-small cell lung cancer, NSCLC）约占85%^[3]^。虽然目前仍没有非常有效的治疗手段，但早期NSCLC的治疗已取得较大的进步，其中以手术为主的综合治疗可以明显延长Ι期NSCLC患者的生存期，5年生存率达到45%-65%^[3]^。因此早期发现肺癌可以减少肺癌的死亡率，但是目前还没有一种非常安全有效的方法对肺癌高危人群进行筛查，以往的肺癌标记物如细胞角蛋白21-1、肿瘤多糖、癌胚抗原诊断肺癌的敏感度较低^[4]^。有研究^[5, 6]^报道影像学检查发现的肺部结节，大约50%是良性的，对减少肺癌死亡率作用有限。此外，超声气管内镜下活检、肺穿刺活检为有创检查方式。

MicroRNAs（简称miRNAs）是一类高度保守、长度很短的非编码调控单链RNA，约20 nt-24 nt，通过与目标mRNA互补配对导致mRNA降解或抑制转录后翻译诱导基因沉默^[7]^。MiRNA参与生命过程中一系列的重要进程，包括发育、造血、器官形成、凋亡和细胞增殖，以及癌症的发生、发展，其通过切割靶mRNA或抑制靶mRNA翻译实现miRNAs抑制基因表达^[8]^。最近研究发现，血清/血浆中有大量稳定存在的miRNA，并且可作为肺癌的诊断标记物。

## 1 血清/血浆miRNA的稳定性

在血清/血浆中存在大量的核糖核酸酶，那么miRNA是否可以逃脱核糖核酸酶的消化？对此Chen等^[9]^、Mitchell等^[10]^2008年报道血清中的miRNA可以耐受核糖核酸酶的消化。Ho等^[11]^报道血浆miRNA在100 ℃的环境下也可保持稳定性。他们将血浆煮沸20 min后提取miRNA，然后测量miRNA的表达量，发现与对照组相比miRNA表达量没有明显变化。此外Taylor^[12]^、Huang^[13]^、Gilad^[14]^等报道在4 ℃和室温下miRNA也可保持稳定性。还有研究^[9-11, 14]^报道miRNA经反复冻融和在强酸（pH=1）、强碱（pH=13）的环境中也可稳定存在。

血清/血浆miRNA保持稳定性的机制仍不清楚，目前主要有两种观点。一种观点认为分泌到血液中的miRNA是以被膜包绕的囊泡状结构存在的，被称之为外核体（exosomes）或微泡（microvesicles），通过此结构可以有效保护血清/血浆中的miRNA不被核糖核酸酶消化，并且可以将miRNA运输到靶细胞，从而发挥调节功能^[15-17]^。另一种观点认为血清/血浆miRNA以蛋白复合体的形式存在^[18]^。Arroyo等^[19]^报道，循环中的miRNA可以被蛋白酶破坏，失去稳定性，进一步研究发现Argonaute2（Ago2）蛋白在该复合体中起着重要作用，因此将循环中的miRNA称之为Ago2-miRNA蛋白复合体。

目前关于血清/血浆miRNA的各种功能机制研究的仍比较少，关于血清/血浆miRNA是如何进入血液、在血液中如何运输以及如何进入靶细胞发挥作用仍不是很清楚，这一系列的问题需要进一步的研究。

## 2 血清/血浆miRNA的检测方法

目前许多研究^[20-23]^通常使用75 μL-1.5 mL的血清/血浆抽提miRNA。Kroh等^[24]^报道用400 μL的血清/血浆抽提miRNA，不仅方便操作，也可抽提出满意的miRNA浓度。目前主要有两种方法提取血清/血浆miRNA，一种是Trizol法，另一种是滤柱法。因Trizol中含有苯酚、异硫氰酸胍等物质，能迅速破碎细胞并抑制细胞释放出的核酸酶，并且能在破碎和溶解细胞时保持RNA的完整性，可以用于直接从细胞或组织中提取总RNA。当前使用最多的是滤柱法，在使用滤柱法时因血清/血浆中含有大量的蛋白质，通常在抽提前需要使用苯酚-氯仿处理样本，以去除蛋白质，防止在抽提过程中大量蛋白阻塞滤柱而大量损耗miRNA。对于检测miRNA的表达量，Northern blot是金标准，但是由于其灵敏度较低并且操作方法较繁琐，一般不用于对临床样本的检测^[25]^。实时荧光定量RT-PCR是检测血清/血浆miRNA表达量的常用方法。根据反转录引物的不同，实时荧光定量RT-PCR可以分为茎环状引物法和多聚腺嘌呤加尾法。研究报道使用茎环状引物法可以获得更高的反转录效率和特异度。此外，根据化学发光原理的不同，还可分为荧光染料法和探针法。探针法在检测的特异度和灵敏度方面优于染料法。此外高通量序列分析法^[28]^和芯片法也用于对miRNA的检测，但由于高通量序列分析法价格较昂贵，一般只用于探寻新的miRNA，而芯片法由于重复性较差，一般只用于miRNA的初筛。

今后随着检测技术的不断发展，需要建立一套标准化的血液样本收集、运输保存以及miRNA提取制备和实时荧光定量RT-PCR检测技术体系以及数据处理系统，确保miRNA检测结果的标准化。

## 3 血清/血浆miRNA在肺癌诊断中的应用

Keller等^[29]^报道肺癌患者的血清miRNA表达谱在确诊为肺癌之前就已发生了改变。在此项研究中共收集到29份血清，其中6份来自6位健康志愿者，另外23份来自已确诊为肺癌的8例患者。在这23份血清中，有15份是在临床确诊为肺癌之前收集到的。他们使用芯片法分析了这29份血清miRNA的表达谱，发现血清miRNA表达谱可以在很长的时间里保持稳定，但越接近确诊为肺癌的时间点，miRNA的表达谱变化越明显。

此外，Boeri等^[6]^将血浆miRNA与螺旋CT对肺癌的早期诊断进行了对比。研究者使用螺旋CT对吸烟者进行了肺癌筛查，并且收集了确诊为肺癌时以及确诊为肺癌之前的血浆。发现当螺旋CT未报告异常时，使用血浆miRNA对肺癌进行诊断，敏感度和特异度分别为80%和90%。而当螺旋CT报告异常时，血浆miRNA对肺癌诊断的敏感度和特异度达到75%和100%，研究还发现某些血浆miRNA与肺癌患者的预后相关。

Chen等^[4]^报道使用10种血清miRNA联合诊断NSCLC的敏感度和特异度分别达到93%和90%。为了获得血清中差异表达的miRNA，研究者使用实时荧光定量RT-PCR技术检测了200例NSCLC患者和110位健康志愿者血清中的miRNA，筛选出10种miRNA，并在另外200例NSCLC患者和110位健康志愿者中对这些血清miRNA的表达差异情况进行了验证。发现使用这10种miRNA联合诊断NSCLC的敏感度和特异度分别为93%和90%。此外他们还使用这10种血清miRNA在2, 000名体检者中进行了NSCLC筛查，发现使用这10种血清miRNA对NSCLC进行筛查，可以得到较好的检出率。

此外，Foss等^[23]^报道血清miR-1254和miR-574-5p可以用于早期NSCLC的诊断，研究者使用芯片技术检测了11例NSCLC患者和11位健康志愿者的血清miRNA表达谱，发现有4种miRNA的差异表达倍数达到了1.54倍。然后在另外的22例NSCLC患者和31位健康志愿者中对这种差异表达情况进行了验证。发现血清miR-1254和miR-574-5p联合诊断早期NSCLC的价值最高，敏感度和特异度达到73%和71%。另外Shen等^[20]^也报道了血浆miRNA-21、miRNA-126、miRNA-210、miRNA-486-5p可以联合诊断Ⅰ期NSCLC。他们通过测定早期肺癌组织中的miRNA表达谱明确了12种miRNA与早期NSCLC有关，然后在28例Ⅰ期NSCLC患者的肺癌组织和血浆中对这些miRNA进行了筛选，并在另外58例NSCLC患者和29例健康志愿者的血浆中对筛选得到的miRNA进行了验证，发现这12种miRNA中有5种差异表达水平在血浆与肺癌组织中是一致的，并且通过*Logistic*回归模型进一步发现有4种miRNA（miR-21、miR-126、miR-210、miR-486-5p）对诊断Ⅰ期NSCLC的敏感度和特异度达到最高（分别为73.33%和96.55%），并且还发现这些miRNA诊断肺腺癌的敏感度（91.67%）明显高于肺鳞癌（82.35%）。

Wei等^[22]^报道血浆miRNA-21可以作为NSCLC的诊断标记物，并且还发现血浆miRNA-21的表达水平与肺癌的TNM分期相关，在T3-T4患者血浆中miRNA-21的表达水平高于T1-T2患者，Ⅲ期-Ⅳ期患者的表达水平高于Ⅰ期-Ⅱ期患者的表达水平。使用血浆miRNA-21诊断NSCLC的敏感度和特异度分别为76.2%和70%。此外，还发现在使用以铂类药物为基础的化疗患者中，达到部分缓解的患者血浆中miRNA-21的表达水平明显低于达到稳定和进展患者的表达水平。

另外Roth等^[30]^报道肺癌患者血清miRNA-10b、miRNA-34a、miRNA-141、miRNA-155的表达水平明显高于健康者，并且还发现肺癌患者血清miRNA-10b、miRNA-141、miRNA-155的表达水平高于肺部良性病变患者的表达水平，肺癌患者血清中miRNA-10b的高表达与肺癌的淋巴结转移相关。

## 4 问题与展望

综上所述，miRNA在血清/血浆中不仅可以稳定存在，并且可以耐受各种极端环境的影响。在其稳定性的基础上，利用它在肺癌患者和健康者中表达谱的不同，可以将肺癌患者筛选出来，以更早地诊断肺癌，降低肺癌的死亡率。但是目前看来，利用血清/血浆miRNA诊断肺癌还存在一个重要问题：血清/血浆中缺少合适的内参基因作为对照。对此已有学者提出疑问^[31]^，他们认为由于缺乏合适的内参基因作为对照，很可能造成实验组与对照组实验数据之间不具有可比性，因此利用血清/血浆中的miRNA诊断肺癌并不合适。此外，由于目前文献报道的用于抽提miRNA的血清/血浆量的不同以及抽提方法和定量方法的不同，造成了相关文献报道的用于诊断肺癌的miRNA不同。因此在后续的研究中希望可以找出合适的内参基因作为对照，并建立起使用血清/血浆miRNA诊断肺癌的标准化程序，促进血清/血浆miRNA在肺癌诊断中的应用。
